# Mortality, morbidity and economic growth

**DOI:** 10.1371/journal.pone.0251424

**Published:** 2021-05-27

**Authors:** Lorenzo Rocco, Elena Fumagalli, Andrew J. Mirelman, Marc Suhrcke

**Affiliations:** 1 Department of Economics and Management, University of Padova (Italy), IZA, Bonn, Germany; 2 Utrecht University School of Economics, Utrecht University, Utrecht, The Netherlands; 3 Centre for Health Economics, University of York, York, United Kingdom; 4 Luxembourg Institute of Socio-economic Research, Esch-sur-Alzette, Luxembourg; University of Genoa, ITALY

## Abstract

The question of whether and how changes to population health impact on economic growth has been actively studied in the literature, albeit with mixed results. We contribute to this debate by reassessing–and extending–[1], one of the most influential studies. We include a larger set of countries (135) and cover a more recent period (1990–2014). We also account for morbidity in addition to mortality and adopt the strategy of providing bounding sets for the effects of interest rather than point estimates. We find that reducing mortality and disability adjusted life years (DALYs), a measure which combines morbidity and mortality, promotes per capita GDP growth. The magnitude of the effect is moderate, but non negligible, and it is similar for mortality and DALYs.

## 1. Introduction

Since the end of World War II mortality rates have been decreasing and life expectancy has been increasing all over the world, with the notable exceptions of the Former Soviet Union during the transition from communism, and Southern Africa during the HIV-AIDS epidemic. However, the extent to which the observed health improvement has played a causal role in promoting the similarly favorable economic development worldwide over the same period remains theoretically ambiguous.

On the one hand, better health may improve economic performance: lower mortality and longer lives would be expected to favor investments in physical and human capital, which in turn are major engines of sustained growth [2,3]. On the other hand, a fall in mortality rates, if not accompanied or preceded by a corresponding fertility decline, accelerates population growth and may lead to lower production per capita [4]. Therefore, quantifying the effect of changes in mortality on economic growth is crucial to helping governments formulate policy to promote or counteract these effects.

A considerable literature has studied the effects of mortality or life expectancy on economic performance. Early studies generally found a strong positive association between health and economic performance, defined as either the growth or level of GDP per capita. More recently, the literature has recognized that this result may be caused by omitted or poorly controlled country characteristics: factors that support or slow down economic development might also be responsible for a country’s population health. For instance, good political and economic institutions may have triggered both sustained economic growth and improved population health by reducing within-country inequalities [5].

The recent empirical evidence on the subject is mixed, as exemplified by the contrasting results of two seminal papers, [4] (Acemoglu and Johnson—AJ) and [1] (Lorentzen, McMillan, and Wacziarg—LMW). While AJ find a negative effect of health on economic performance, LMW find a positive one despite the fact that both studiesuse broadly comparable specifications. AJ exploit the longitudinal nature of a panel of low and middle-income countries (LMIC) between 1940 and 1980 and set up a first-difference model where differenced GDP per capita is regressed on differenced life expectancy (their proxy of health). They find that improvements to life expectancy have a significant negative effect on GDP per-capita. LMW define a cross-sectional growth regression model and regress differenced GDP per capita on the initial level of GDP per capita and health. They consider adult and infant mortality rates as iesproxies of poor health in a global sample of over 80 countries between 1960 and 2000 and find a strong significant negative effect of both adult and infant mortality on per-capita GDP growth.

Subsequent papers tried to reconcile the AJ and LMW results. Particularly, [6] reconsidered the AJ analysis, and by separating the sample into pre- and post-demographic transition countries, it was able to replicate the AJ findings of negative growth impacts only for the former group of countries (characterized by high mortality, high fertility and increasing population growth). More recently, [7] indirectly confirmed this result, finding a positive effect of life expectancy both on GDP and GDP growth on a small sample of post-demographic transition countries.

Complementing [6], in this paper we reconsider LMW analysis and we extend it along several dimensions. First, we use more extensive and more recent data studying the impact of mortality rates on long-run GDP per-capita growth in a sample of 135 countries between 1990 and 2014. Second, we assess the impact of disability-adjusted life years (DALYs) per capita (a combined measure of morbidity and mortality) in the working age population, besides population mortality: this helps us to assess the long-term impact on human capital and productivity. Third and more importantly, methodologically we take a different stand compared to the literature. We aim to provide bounding sets for the effect of population health on economic growth, to give the reader a range of plausible estimates rather than a unique value. This is because we share the concern for the endogeneity problems which are responsible for possibly large bias in the estimates and threaten the causal interpretation of the findings. Particularly concerning is the validity of the instrumental variables (IV) used in the literature. The predicted mortality instrument used in AJ has been criticized by [8] and [9], both on the grounds of its relevance and of its validity. Similarly, the battery of geographical, climatic and epidemiological conditions exploited by LMW raises aa concern that these are structural characteristics which shaped a country’s economy, its culture and social organization, and not only population mortality. Lacking better alternatives and withstanding the importance of answering the question of whether population health is a lever of economic growth, we take the more prudent approach of providing credible bounding sets for the effects of interest, rather than point estimates.

We first assess to what extent the omitted variable bias drives the association between GDP per capita growth and mortality, by applying the strategy suggested by [10]. We find that if some variables were omitted from the model, the estimated effect of mortality and morbidity on GDP per capita would actually be larger in absolute value. We put under scrutiny this “optimistic” conclusion by performing an IV analysis, where we instrument mortality (resp. DALYs) by the proportion of a country’s inhabitants exposed to the Plasmodium Falciparum in 1966. This is a (pre-determined) contributor of Sachs’ Malaria Ecology Index that was part of the original LMW set of instrumental variables which is, we argue, the least criticizable among the instruments in LMW.

Contrary to geographical and climatic conditions which are persistent in the long run and likely shape a country development path, the spread of malaria varied over time quite substantially, and its diffusion in 1966 only partially corresponds to its spread during our study period and to its diffusion in ancient times. Accordingly, the concern that malaria determines the evolution of a country economy, as geographic and climate variables can do, is less compelling. Historically, malaria was common in the US, Europe and Russia. For instance, poet Friedrich Schiller contracted the disease in Germany, Oliver Cromwell in Ireland, and Abraham Lincoln in Illinois [11]. In Italy, Spain and Northern Australia malaria was prevalent in the low lands until the Fifties and the Sixties respectively. The marked variation of malaria prevalence overtime is also apparent from its death toll, which reached its peak around 2005 to over 800k deaths per year and thereafter declined by more than one quarter. Malaria diffusion responds to the implementation of public health measures, especially the widespread use of insecticides, the drainage of swampland for expanding agricultural land, accessibility to treatments and improvements in housing conditions. In the tropical and sub-tropical areas, in Africa, Southern Asia and Central and Latin America, malaria is still endemic but on retreat. Also, the spread of malaria varies within countries, low and humid lands being more prone than others.

Both rich and poor countries today, were in the past affected by malaria, nonetheless we cannot rule out the possibility that malaria is correlated with a country’s economic performance throughout mechanisms other than its population health or mortality. For instance, malaria can slow down the economic development of low and humid areas, and force the population to concentrate on high lands, distant from rivers and other natural communication routes. If so, our IV might fail to meet the exclusion restriction and produce biased estimates.

To address this concern, we adopt a battery of methods, most of which belong to the domain of partial identification, including [12,13] and [14] to provide bounds and check how robust are our estimates to the violation of IV assumptions. To the best of our knowledge this is the first time such methods have been employed in this literature.

We confirm that both mortality and morbidity have a negative effect on GDP per capita growth. The effect of reducing mortality by 10 percent is that of adding *at least* 9.6 percentage points to GDP per capita growth over a period of about one quarter century, according to [13] bounding strategy. These estimates are fully coherent with the bounds obtained under [10] assumptions. The effect of an equivalent reduction in DALYs is comparable to that of mortality. According to [13] the effect of reducing morbidity by 10 percent is that of increasing long run growth of *at least* 10.6 percentage points. Importantly, our IV estimates are robust to [12] and [14] stress tests.

We also find that the effects of mortality and morbidity are heterogenous across countries, with the low and the high income countries displaying a stronger effect than the middle income countries. Similarly, the effect varies non-linearly depending on the stage of the demographic transition at the beginning of the study period.

Finally, we illustrate the expected impact in monetary terms of reducing or increasing mortality and morbidity in four selected countries, representing high income (the US), upper-middle income (China), lower-middle income (India) and low-income countries (Niger). Specifically, we simulate the effect of marginally increasing or decreasing a country’s commitment to health policies effective in reducing mortality and morbidity. We find non-negligible benefits (costs) in strengthening (weakening) health policies, especially so in Niger and in the US.

The rest of the paper is organized as follows. Section 2 briefly reviews the relevant literature. Section 3 describes the data used. Section 4 introduces the methods adopted. Results are reported in Section 5. The economic significance of our findings is discussed in Section 6 and conclusions follow.

## 2. Previous literature

Early studies generally found a strong positive association between population health and economic growth. For instance, [15] reports that life expectancy is among the most robust predictors of GDP per-capita growth. However, as [16] pointed out, much if not all of the early work on the subject “suffer(s) from severe problems of endogeneity and omitted variable bias” ([16], p.1271).

The more recent literature explicitly acknowledges endogeneity problems and tries to uncover the causal effect of health on GDP growth (see [3], for a review). Two seminal papers have arguably renewed the interest in this question and have often been contrasted against one another due to their contrasting conclusions. The first is AJ, which adopts an IV strategy coupled with country fixed effects to analyze the influence of life expectancy on income levels. Its identification strategy rests on the discovery of new chemicals and drugs to fight communicable diseases. This IV approach combines the timing of discoveries with the country-specific prevalence of the diseases that the new drugs are meant to treat. AJ covers 47 low and lower middle-income countries for which data on total GDP, per-capita GDP and life expectancy are available in 1940, 1980 and 2000. The results indicate that life expectancy has no significant effect on total GDP level, a significantly positive effect on population size and, hence, a negative effect on per-capita GDP.

The second is LMW, which also uses an IV strategy, this time to analyze the effect of mortality rates on per-capita GDP growth. Their sample covers 88 countries, and per-capita GDP growth between 1960 and 2000 is regressed on the average adult and infant mortality rates registered between 1960 and 2000, the log of per-capita GDP in 1960, and several country controls. Mortality rates are instrumented by the Malaria Ecology Index developed by [17] and a battery of climatic variables and geographic characteristics. The results indicate a strong negative (i.e. health-improving) and significant effect of both adult and infant mortality rate on per-capita GDP growth.

AJ and LMW address similar questions, both adopt IV identification strategies, and have comparable model specifications. Unsurprisingly, the divergence in their findings has triggered a substantial research effort in more recent years. [18] points out that the AJ model omits the initial level of life expectancy. When the authors estimate a model similar to LMW, but with per-capita income growth between 1960 and 2000 regressed on the change in life expectancy in the period and its initial level, they find a positive contribution of both variables on growth. A similar point has been raised by [9]. This paper also notes that controlling for initial life expectancy largely reduces the strength of AJ’s instrument, which is highly correlated with mortality rates in the 1940s. Recently, [19] extends the AJ dataset by adding an observation relative to year 1900 for 35 of the original 47 countries. Hence two long term differences for each country are computed, one between 1900 and 1940 and one between 1940 and 1980. With two observations per country, country fixed effects can be included in the first-difference model. Therefore, [19] accounts for the criticism raised by [9], that initial life expectancy was omitted from the AJ model. Reassuringly, the results confirm and reinforce AJ conclusions.

[20] develops an estimation strategy akin to AJ’s to estimate the effect of life expectancy on per-capita GDP across US states between 1940 and 1980. In line with AJ, the adopted instrument is the interaction between mortality from 9 medically treatable communicable diseases registered before discovering antibiotics and the date antibiotics were discovered. The advantages of this within-country analysis are first, in the comparison of relatively homogenous economic systems, and second, in the expected smaller measurement error due to the accurate vital registration system established in America since 1933. The results show that increased life expectancy has significant positive effects on population level and total GDP, while the effect on GDP per capita is small and statistically insignificant.

In an attempt to reconcile the AJ and LMW results, [6] replicate the AJ analysis on the same data but distinguish between countries that, as of 1940, had already completed their demographic transition towards a low fertility and low-mortality regime and countries that had not. The key observation of [6] is that the introduction of new pharmaceuticals had the immediate effect of reducing mortality, which was not immediately accompanied by a reduction in fertility. It follows that, at least for a certain period, population grew faster, causing a fall in income per capita. The authors alternatively use the AJ and LMW instruments and, in both cases, they find that increasing life expectancy causes an increase in GDP in post-transition countries while the effect is generally negative, though not always significantly different from zero, among pre-transition countries. They conclude that the opposing results in AJ and LMW are not due to the alternative instrumentation strategies, but rather to differences in the samples of countries and time periods examined. In a later short paper [21], the same authors further increase the degree of flexibility of their empirical analysis by defining a mixture model that allows the data to determine whether any given country is to be considered pre- or post- demographic transition rather than relying on an ex-ante classification.

From a perspective close to that of [6,22] notes that the response of GDP per capita to health depends on what health dimension is considered. Whether health shocks mainly affect adults or children makes a difference for the magnitude and the timing of the effects. In the former case the effect is mainly through a variation in population size, while in the latter case it is mainly through a variation in investments in human capital and indeed in the future supply of skilled labour.

An interesting source of exogenous variation has been exploited by [23]. They instrument health (captured by mortality rates) by means of the progressive introduction of universal public health care in 12 European countries between 1820 and 2010. Their findings indicate that the introduction of public health systems led to significant reductions in infant mortality and crude death rates that, in turn, had a significant positive effect on economic growth.

On the whole, the existing evidence appears to support the hypothesis of a positive effect of longer life expectancy and lower mortality on economic growth. However, the existing work tends to be limited to small samples of countries or focus on particular regions. Using larger samples, as we do in this paper, allows us to achieve better global coverage and to harness the empirical advantages of a larger sample size.

## 3. Data

The data used in this paper come from different sources. We consider population all-cause mortality (per 100,000 inhabitants) and DALYs (per 100,000 inhabitants) in the working age population (aged 15–64 years). DALYs are defined as the sum of years of potential life lost due to premature mortality and the years of productive life lost due to disability. While DALYs in the working age population mainly captures the influence of health on economic growth mediated by labour supply and productivity, population mortality also accounts for the macroeconomic effects of the survival rates in the elderly population, through savings, investment decisions and social security expenditures, among others. Mortality and DALYs data are from the Institute for Health Metrics and Evaluation (IHME) Global Health Data Exchange (GHDx) GBD results tool. Mortality data are available annually for the timespan 1990 to 2015 while DALY information is only available every fifth year. In the appendix we replicate the baseline analysis with mortality in the working-age population and DALYs in the whole population, to assess the sensitivity of our findings to age-restrictions.

Data on malaria, and Köppen-Geiger climatic zones are from the Harvard University’s Center for International Development (CID) database. We aggregate the original Köppen-Geiger climatic zones in six indicators of the proportion of the country area in polar, boreal, humid temperate, dry temperate, subtropical and tropical zones. Version 9.0 of the Penn World Tables (PWT) provided data for real GDP as well as for government consumption, exports and imports (in constant 2011 national prices using United States Dollars [USD]). The World Bank’s World Development Indicators (WDI) database provides data on population, the share of population aged 65 and more, urbanization (% population living in urban agglomeration greater than 1 million population) and internet penetration. The population share achieving at least secondary education is obtained from the updated Barro-Lee (BL) world dataset (2012) and a measure of social capital, namely the average level of generalized trust in the population, is obtained from the World Values Survey (WVS). Finally, the 2002 version of the Freedom in the World (FIW) database provided an index of political rights and civil liberties.

Overall, we have complete information for 135 countries. We compute the per-capita GDP growth rate between 1990 and 2014 (in percentage points) and we average the remaining variables over the period 1990–2014, by country. Key descriptive statistics are reported in Table 1. In the sample, income per capita increases on average by 63 percent from its 1990 level. Average mortality rate in the population is 896.14 per 100,000 inhabitants per year and the number of healthy years lost for disability in the working age population is 18,630 per 100,000 inhabitants on average.

**Table 1:** Summary statistics. Variables Averaged over the period 1990–2014.

Variable	Obs	Mean	Std. Dev.	Min	Max
Real GDPpc growth 1990–2014 (percent)	135	64.13	65.90	-43.68	441.96
All-cause DALYs (years per 100,000 inhab.) all ages	135	44.81	24.33	15.14	116.20
All-cause DALYs (years per 100,000 inhab.) 15–64	135	18.63	6.72	9.34	44.44
All-cause Mortality Rate (deaths per 100,000 inhab.) all ages	135	896.14	334.76	254.77	1812.25
All-cause Mortality Rate (deaths per 100,000 inhab.) 15–64	135	483.18	280.21	161.47	1407.69
Falciparum Malaria Index 1966 (% of resident population at risk)	135	32.65	42.47	0.00	100.00
log Real GDPpc 1990	135	8.72	1.19	5.90	11.70
Share of population aged 65 and over in 1990	135	6.36	4.22	1.20	17.82
OECD 1990	135	0.14	0.35	0.00	1.00
Log population	135	16.39	1.37	14.06	20.97
Urban (% of urban population)	135	54.33	22.11	8.82	100.00
Openness index	135	75.87	38.92	17.61	322.93
Government spending (% of GDP)	135	17.15	11.27	3.68	101.75
Civil liberties (Freedom House Index)	135	3.72	1.68	1.00	7.00
Internet (% population with internet access)	135	18.05	15.60	0.47	56.44
Share of population with at least secondary education	135	47.94	25.71	0.00	95.73
Share of population reporting they trust other (WVS)	135	24.26	15.02	3.50	73.90
Trust information missing	135	0.32	0.47	0.00	1.00
Proportion area in polar zone	135	0.02	0.06	0.00	0.30
Proportion area in boreal zone	135	0.06	0.16	0.00	0.91
Proportion area in dry temperate zone	135	0.04	0.13	0.00	0.95
Proportion area in wet temperate zone	135	0.20	0.32	0.00	1.00
Proportion area in subtropical zone	135	0.29	0.33	0.00	0.98
Proportion area in tropical zone	135	0.17	0.26	0.00	1.00

## 4. Methods

To estimate the effect of mortality and morbidity on economic development we estimate a long run growth regression model akin to LMW. A long-run growth model relates long-run per-capita GDP growth (over the period between 1990 and 2014, the last year for which all the information is available) to initial income and, alternately, the average mortality rate and the average DALY over the same period. Formally the model is specified as follows:

$$g_{i}=\pi_{0}+\pi_{11}\log(y_{i1990})+\pi_{12}pop65_{i1990}+\pi_{2}{\bar{R}}_{i}+\pi_{4}{\bar{X}}_{i}+\pi_{5}W_{i}+\varepsilon_{i}$$ (1)

where $g_{i}=\frac{y_{i2014}-y_{i1990}}{y_{i1990}}$ is the long run growth rate of real per-capita GDP in country *i* from 1990 to 2014, log(*y*_*i*1990_) is the logarithm of per-capita GDP in 1990, the initial condition, *pop*65_*i*1990_ is the share of the population aged 65 and more in 1990, that we interpret as an indicator or the stage of the demographic transition at the beginning of the period, ${\bar{R}}_{i}$ is respectively the average mortality or DALY between 1990 and 2014, ${\bar{X}}_{i}$ is a vector of time-varying country controls averaged over the period 1990–2004 and *W*_*i*_ a vector of time-invariant controls.

Time-varying controls include log population, proportion of urban residents, proportion of residents with internet access, government spending, and an index of openness to the global economy (defined as the ratio between the sum of imports and exports and national GDP), human capital (proxied by the share of population with at least secondary education) and social capital (the share of population reporting that their trust the others in the WVS). Time-invariant controls include a set of variables accounting for the proportion of the country area in polar, boreal, humid temperate, dry temperate, subtropical and tropical zones, and two proxies of institutional quality (an index of civil liberties and the condition of being a member of the OECD in 1990).

Error terms of neighboring countries may be correlated. As a robustness check we estimate the same regression by clustering the standard error at the macro-regional level (i.e., East Asia & Pacific, Europe & Central Asia, Latin America & Caribbean, Middle East & North Africa, North America, South Asia, Sub-Saharan Africa) to account for possible correlation of the error terms within macro-regions, due to the interlinked economic dynamic of neighboring countries. Due to the limited number of clusters, we bootstrap the standard errors using wild bootstrap.

The key explanatory variable is ${\bar{R}}_{i}$ and the parameter of interest is its marginal effect *π*_2_. Variable ${\bar{R}}_{i}$ is likely endogenous for three reasons. First, ${\bar{R}}_{i}$ can be correlated with unobservable country characteristics omitted from the model. Particularly, the usual catching-up of the less developed towards the more developed economies is only partially accounted for by the inclusion of initial income in 1990 in a long run model that spans over two decades. Second, ${\bar{R}}_{i}$ could suffer from measurement error [1]. Third ${\bar{R}}_{i}$ could suffer from reverse causation, because during periods of economic growth social interactions and the spread of viruses and communicable diseases increase [24], lifestyles change, housing and working conditions improve, access to healthcare services expands, although stress and road traffic injuries escalate (see [25] and [3], for an ampler account).

To address endogeneity, we use three complementary strategies. First, we acknowledge that we may fail to account for some unobservables. Following [10], we compute the size of the coefficient we would get if the selection on unobservable was accounted for. As in [10], we assume that unobservables are as important as observables in explaining long run growth, and that their introduction improves model explanatory power (i.e. the R^2^) by respectively 30 percent and 50 percent. Under these conditions we estimate the resulting parameter *π*_2_. Our estimates indicate that the correlation between ${\bar{R}}_{i}$ and ε is positive and the OLS estimates are upward biased (i.e. they are negative, but smaller in absolute value, compared to the plausibly unbiased estimates). In other words, the selection bias makes the effect estimated by OLS conservative.

Second, we adopt an instrumental variables (IV) strategy to put under scrutiny the finding that OLS estimates are conservative. While LMW instrument ${\bar{R}}_{i}$ by the Malaria Ecology Index (a measure of predicted mortality due to malaria in 2004 defined by [17]) and a series of climatic and geographic characteristics, we opt for a just identified two-stages least squares estimator (2SLS). ${\bar{R}}_{i}$ is instrumented with the Malaria Falciparum Index prevailing in 1966 (Z), i.e. the proportion of the population of 1966 exposed to Plasmodium Falciparum, the most prevalent type of malaria, especially in Africa. We believe that the 1966 Malaria Falciparum Index is preferable over the Malaria Ecology Index because it is based on data referring to a time largely preceding our study period and does not depend on the malaria protection policies implemented by the countries in the recent decades.

Differently from LMW, we add the proportions of the country surface belonging to the Köppen-Geiger climatic zones to the controls, instead of including them in the set of instruments. We take this stand for two main reasons. First, climatic zones may have a direct effect on GDP growth, because they shape agricultural productivity and likely contribute to set the path of long run economic development. Second, overidentified models with many instruments tend to exacerbate the finite sample bias of IV estimators, unless all instruments are strong enough to keep high the first stage F statistics ([26], p.209). Unfortunately, this is not the case with LMW instruments, as it is evident from their first stage results (Tab 6 in LMW).

In our analysis identification depends on the variation of the Malaria Falciparum Index for given distribution of climatic zones in a country. Such variation is ample because in most countries we observe a plurality of climatic zones. Only two countries of the sample belong to a unique climatic zone, and only in 24 cases out of 135, one climatic zone covers more than 90 percent of the surface. Moreover, while the prevalence of malaria is high in tropical and subtropical areas, not all countries which are prevalently tropical and subtropical are equally affected by malaria, the spread of which depends on land elevation, humidity, presence of water pools and the effectiveness of past eradication campaign. Similarly, while malaria is less prevalent temperate areas, about one third of the countries mainly located in temperate regions were affected by malaria, to different extents, in 1966.

An instrumental variable is valid if it satisfies the conditions of relevance (i.e., the instrument is correlated with mortality/morbidity) and excludability (i.e., the instrument does not directly affect GDP growth). The first requirement is testable, and we show that our instrument is strongly correlated with mortality and DALYs. The excludability condition is untestable, at least in a just identified model. We are concerned that the instrument may affect GDP growth via its correlation with other country characteristics. First, malaria prevalence may be correlated with climate features that can directly affect growth: to partly neutralize this channel we condition on climatic zones. Second, malaria prevalence may be high in Sub-Saharan Africa, a region that historically had low economic growth due to poor institutions and a recent past of colonial domination [5]. To address this concern, we condition on measures of institutional quality such as the index of civil liberties (derived from the Freedom House), the status of member of the OECD in 1990 and internet diffusion as a proxy of access to information. We acknowledge, however, that despite these controls, malaria can have influenced the process of economic development through alternative mechanisms, including the kind of productive specialization and the level of transportation and transaction costs.

For these reasons we apply two methods of partial identification proposed by [12] and [13] and we use generated heteroskedasticity-based instruments proposed by [14]. [12] and [13] derive bounds for the coefficients for small deviations from the excludability condition i.e., if we assume that the instrument has a (small) direct effect on GDP growth; [14] suggests alternative instruments that can help identification as they meet the excludability condition by construction.

Following [12] we compute what is the largest direct effect compatible with a statistically significant effect of mortality on economic growth. [12] suggests two methods to achieve this purpose. The first assumes a plausible range of variation for the direct effect. For each value of the direct effect, we can compute a different value of the marginal effect π_2_. The confidence intervals of π_2_ are computed as the union of the confidence intervals obtained for each possible value taken by the direct effect (union of confidence interval approach). The second method assumes that the coefficient of the direct effect is a random variable with a probability distribution (local to zero approach). Explicit bounds can be obtained under the assumption that the direct effect follows a Gamma distribution.

[13] shows that it is possible to obtain informative bounds on the true value of π_2_ even if the instrument has a direct effect on the GDP growth, provided that the sign of the correlation between the endogenous variable ${\bar{R}}_{i}$ and the error term ε is the same as the sign of the correlation between the instrument Z and error term ε. We have mentioned above that the correlation between ${\bar{R}}_{i}$ and ε is positive. We suggest that also the correlation between exposure to malaria falciparum (Z) and ε is positive. [13] shows that if the correlation between ${\bar{R}}_{i}$ and ε has the same sign as the correlation between Z and ε, then π_2_<min[π_2_^IV^, π_2_^OLS^].

[14] proposes an approach to generate additional instruments to use in combination with Z. Such instruments are created using a subset V of the controls in (1) i.e., a subset of the vector [$\bar{X}$, *W*]. For each variable in V, we can construct a new instrument *Z*^*L*^ = (*V*–*E*(*V*))*θ* where *θ* is the error term of the regression of ${\bar{R}}_{i}$ on $\bar{X}$ and W, and *E*(*V*) is the average of V in the sample. Under exogeneity of the controls in V and heteroscedasticity of *ε*, the generated instruments *Z*^*L*^ are correlated with ${\bar{R}}_{i}$ and uncorrelated with *ε*. Therefore, they met the excludability condition and are valid. Adding the generated instruments to Z makes the model overidentified and allows to perform an overidentification test to assess Z validity. We adopt this procedure in two polar manners. First, to keep the IV finite sample small, we add only one strong generated instrument [26]. Hence, we specify *V* as the percentage of the country in tropical or subtropical areas, which is strongly correlated with ${\bar{R}}_{i}$. Second, to avoid any arbitrariness in the specification, we make *V* coinciding with the full set of controls, i.e. V = [$\bar{X}$, *W*]. This maximizes the number of overidentifying restrictions, but the IV finite sample bias is expected to be large in this case, despite all additional instruments are exogenous by construction.

## 5. Results

Baseline results are reported in Table 2 (and fully displayed in Table A1). The upper panel refers to mortality and the lower panel to DALYs. In column 1 we report OLS estimates of Eq (1). Clustering the standard errors at the regional level, to account for possible interdependences between countries performances, makes the estimates only marginally less precise (see Table A2). Following [10], we investigate the bias due to possible unobservables, under the assumption that unobservables and observables are equally correlated with mortality (resp. DALY). This assumption corresponds to a conservative scenario according to [27] and [10]. We observe that removing the selection bias increases (in absolute value), rather than offsetting, the estimated effect of mortality and DALYs on economic growth, which would fade out only under the implausible assumption that included controls and omitted variables are correlated with mortality/morbidity in opposite ways. Assuming that the inclusion of unobservables increases model explanatory power by, respectively 30 percent (column 2 of Table 2) and by 50 percent (column 3), we find that the bunding sets for the effect of population mortality on economic growth, expressed as semi-elasticities, are respectively [-1.048,-0.802] and [-1.317,-0.802]. Reassuringly, these bounding sets are rather narrow and reveal that reducing mortality by 10 percent increases the long run economic growth by between 8 and 13 percentage points. The corresponding bounding sets for the effect of DALY on economic growth are about half as large: reducing DALYs per capita of 10 percent adds between 4.9 and 6.4 percentage points to the long run economic growth. We judge the size of these effects as moderate if compared with the GDP per-capita growth rate of US (41.23 percent), China (441.96 percent), India (207.2 percent) and Niger (7.06 percent), in the period.

**Table 2:** The effect of mortality/DALYs on GDPpc growth. Long-Run Growth Regression.

	(1) OLS	(2) Oster’s (2019) estimate (Rmax = 1.3* R^2^)	(3) Oster’s (2019) estimate (Rmax = 1.5* R^2^)
All-Cause mortality rate (per 100,000 inhabitants)	-0.090***	-0.117	-0.147
	(0.022)		
Semi-elasticity	-0.802	-1.048	-1.317
R^2^ / Rmax	0.498	0.647	0.747
All-Cause DALYs (x 100 inhab.)	-2.648 **	-3.069	-3.412
	(1.017)		
Semi-elasticity	-0.493	-0.572	-0.636
R^2^ / Rmax	0.448	0.582	0.672
Observations	135	135	135

Note: Robust standard errors in parentheses.

*** p<0.01,

** p<0.05,

* p<0.1. Controls are: Log real GDP per capita 1990, share of the population aged 65 and over in 1990, OECD 1990, log population, dummy indicator of rural areas, government spending, civil liberty index, percentage of household with internet access, share of population with at least secondary education, share of population reporting their trust the others in the WVS, dummy indicating whether the trust variable has been imputed, % of country area in polar zone, % of country area in boreal zone, % of country area in temperate zone, % of country area in subtropical zone, % of country area in tropical zone. Semi-elasticity is defined as the effect on economic growth (measured as percentage points) of reducing mortality by 1 percent at the sample mean.

Comparing column 1 with either columns 2 or 3, we conclude that OLS estimates are upward biased (less negative than they should be), implying that the correlation between ${\bar{R}}_{i}$ and the error term ε is positive. Hence mortality and morbidity are higher in countries that have a larger potential for growth unexplained by the controls in model (1). We conjecture that part of this positive correlation can be attributed by the usual catching-up effect. A similar result was detected in LMW and was ascribed to measurement errors by the authors.

IV estimates support the conclusions of the test proposed in [10] and confirm that OLS estimates are conservative (see Table 3). Indeed, IV estimates are twice as large in absolute value than OLS for DALYs, and are marginally larger for mortality. The gap between OLS and IV estimates in the case of DALYs may signal that the OLS is underestimated because of reverse causation. Indeed, morbidity increases during economic expansion due to the spread of communicable diseases, increased stress, and road traffic accidents, which might hamper labour supply and productivity, without impacting mortality appreciably (see [24]). The Kleibergen-Paap rank test displays a F statistic of 58.066 and 24.535 in the case of mortality and DALY respectively, providing evidence that our instrument is strong.

**Table 3:** The effect of mortality/DALYs on GDPpc growth. Reduced form and first stage.

	(1) IV	(1) OLS: reduced form	(2) Oster’s (2019) estimate (Rmax = 1.3* R^2^) Reduced form	(3) OLS: First stage	(4) Oster’s (2019) estimate (Rmax = 1.3* R^2^) First stage
All-Cause mortality rate (per 100,000 inhabitants)	-0.096 ***	-52.462**	-56.669	545.979 ***	357.4535
	(0.037)	(22.592)		(72.591)	
Semi-elasticity	-0.861				
R^2^ / Rmax	-	0.4340	0.5714	0.6953	0.9039
First stage Kleibergen-Paap F test on excluded instruments	58.07				
All-Cause DALYs (x 100 inhab.)	-5.687 **	-52.462**	-56.669	9.225***	6.28446
	(2.488)	(22.592)		(1.866)	
Semi-elasticity	-1.060				
R^2^ / Rmax	-	0.4340	0.5714	0.5012	0.6516
First stage Kleibergen-Paap F test on excluded instruments	24.535				
Observations	135	135	135	135	135

Note: Robust standard errors in parentheses.

*** p<0.01,

** p<0.05,

* p<0.1. Controls are: Log real GDP per capita 1990, share of the population aged 65 and over in 1990, OECD 1990, log population, dummy indicator of rural areas, government spending, civil liberty index, percentage of household with internet access, share of population with at least secondary education, share of population reporting their trust the others in the WVS, dummy indicating whether the trust variable has been imputed, % of country area in polar zone, % of country area in boreal zone, % of country area in temperate zone, % of country area in subtropical zone, % of country area in tropical zone. Semi-elasticity is defined as the effect on economic growth (measured as percentage points) of reducing mortality by 1 percent at the sample mean.

**Fig 1:**
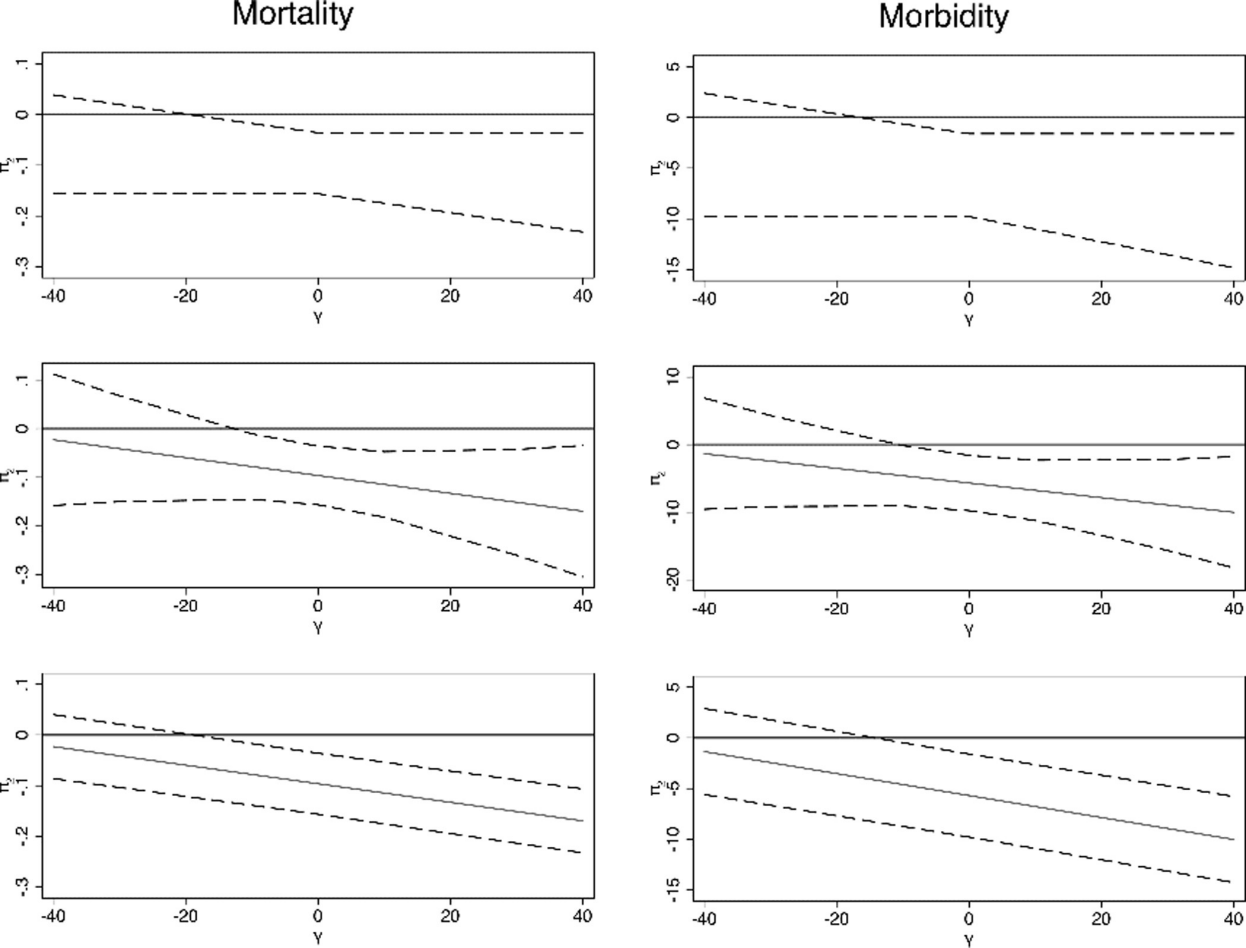
A. Small violations of the exogeneity assumption on the effect of mortality (Conley et al. 2012). B. Small violations of the exogeneity assumption on the effect of DALY (Conley et al. 2012). Note: upper panel: union of confidence interval approach; middle panel: local to zero approach with variance equal to the square of the mean; lower panel: local to zero approach with variance equal to the mean. π_2_ is the real causal effect of mortality on GPD per capita growth and γ is the direct effect of the instrument on GDP per capita growth.

IV estimates could be inflated if malaria affected economic activity not only indirectly, through increased mortality and morbidity, but also directly by hampering economic activities. We scrutinize the stability of our IV estimates by relaxing the assumption of exogeneity of the instrument. We start by considering Conley’s test [12]. Results are reported in Fig 1A for mortality rates and 1B for DALYs. In all cases we report the 90-percent confidence intervals associated to π_2_ under various hypothesis regarding the distribution of γ, i.e. the direct effect of Z on *g*_*i*_. In the first panel, we adopt the union of confidence intervals method, and set γ between -40 and +40. In the second panel and third panel we assume that γ follows a Gamma distribution with respectively mean μ between -40 and +40 and variance equal to μ^2^. Results indicate that π_2_ would be statistically insignificant if the direct effect γ was (approximately) smaller (more negative) than -15. We consider this case unlikely because it would imply that γ would be larger than one fourth of the value of the total effect of Z in the reduced form equation (i.e., -52.46), which accounts for both the direct effect of Z and that mediated by the endogenous variable. For any value of γ> -15, our conclusions on the size of the real causal effect π_2_ would be either qualitatively confirmed or reinforced.

When the instrument exclusion restriction is violated, [13] show that the true causal effect π_2_ must be smaller than both the IV and the OLS estimates, that is π_2_<min[π_2_^IV^, π_2_^OLS^], if the correlation between the ${\bar{R}}_{i}$ and the error term ε has the same sign as the correlation between the instrument Z and the error term ε. Since π_2_^IV^ < π_2_^OLS^ (Table 2), corr (${\bar{R}}_{i}$, ε) must be positive. We show that corr(Z, ε) is also positive by means of a two-stage Oster’s test [10]. More specifically, we apply the method in [10] separately on the reduced form and the first stage corresponding to model (1) and assess how unobservables influence a) the correlation between Z and *g*_i_, and b) the correlation between Z and ${\bar{R}}_{i}$. It turns out that the inclusion of unobservables slightly increases in absolute value the reduced form coefficient while it largely shrinks the coefficient of first stages, preserving their sing (Table 3). Therefore, accounting for unobservables produces even more negative IV estimates (recalling that the IV estimate is the ratio between the reduced form and the first stage parameters in a just identified model) and implies that corr(Z,ε)>0. We conclude that the true causal effects are bounded from above by our IV estimates, and they satisfy *π*_2_ <-0.096 for mortality (semi-elasticity<-0.861), and *π*_2_ <-5.686 for DALYs (semi-elasticity<-1.060).

To further assess the robustness of our IV estimates, we add to our model the Lewbel’s generated instruments [14] in combination with Z. We start with the most parsimonious specification where we add only one generated instrument, the one derived from the proportion of county located in the tropical and subtropical climatic regions. We test instrument relevance, and we find that instruments are strong, as the Kleibergen-Paap rank test yields an F statistic of 45.87 (resp. 18.38) in the mortality (resp. DALY) first-stage regression. The Hansen overidentification test displays a J statistic of 0.372 (p = 0.5419) in the model of mortality, and 0.320 (p = 0.5719) in that of DALYs and does not reject the null hypothesis of instrument’s exogeneity. The coefficients π_2_ in both regressions (column 3 of Table 4) are close to those obtained via our baseline IV specification (column 2) and are statistically significant at conventional levels. With the richest specification, where we add as many instruments as are our controls in model (1), the F statistics of Kleibergen-Paap rank test turn to be lower, especially so for DALYs, indicating that some of those instruments are weak (F = 18.735 for mortality and F = 10.349 for DALYs). The Hansen J statistic are 21.289 (p = 0.2651), for mortality and 25.485 (p = 0.1121) for DALYs. Point estimates remain stable for mortality and get smaller for DALYs. As to be expected with many weak instruments [26], the latter estimates are likely biased towards the OLS.

**Table 4:** The effect of mortality/DALYs on GDPpc growth. Long-Run Growth Regression estimated adding Lewbel’s (2012) heteroskedasticity based instruments.

	OLS	IV	IV Lewbel (2012) (V = country area in the tropical and subtropical region)	IV Lewbel (2012) (V = all controls)
**All-Cause mortality rate (per 100,000 inhabitants)**	-0.090*** (0.022)	-0.096 *** (0.037)	- 0.089 *** (0.031)	-0.094*** (0.028)
Semi-elasticity	-0.802	-0.861	-0.795	-0.841
**All-Cause DALY (per 100 inhabitants)**	-2.648 ** (1.017)	-5.687 ** (2.488)	-4.820** (2.145)	-2.639* (1.351)
Semi-elasticity	-0.493	-1.060	-0.898	-0.492

Note: Robust standard errors in parentheses.

*** p<0.01,

** p<0.05,

* p<0.1. Controls are: Log real GDP per capita 1990, share of the population aged 65 and over in 1990, OECD 1990, log population, dummy indicator of rural areas, government spending, civil liberty index, percentage of household with internet access, share of population with at least secondary education, share of population reporting their trust the others in the WVS, dummy indicating whether the trust variable has been imputed, % of country area in polar zone, % of country area in boreal zone, % of country area in temperate zone, % of country area in subtropical zone, % of country area in tropical zone.

We finally investigate possible heterogeneous effects by distinguishing between countries classified as low, lower-middle, upper-middle and high income according to the World Bank income group classification as of 2014. We add to model (1) the interaction between ${\bar{R}}_{i}$ and four dummies, each indicating one class of countries. We also study how effects vary with the share of population aged 65 and above in 1990, an indicator of the stage of demographic transition at the beginning of the period. We cut this variable in three intervals, below 5 percent, between 5 and 10 percent and above 10 percent. We group countries accordingly, and each group corresponds to a dummy variable that we interact with ${\bar{R}}_{i}$. Most countries cluster in the first interval and among them there is little variation in the share of the senior population. The remaining countries are evenly distributed across the two remaining intervals. We remark that there is not perfect overlapping among the country grouping by income and by share of the senior population. Among countries where the share of the senior population is less than 5 percent, we find most low income countries besides several rich and middle income countries in the Gulf area and Eastern Asia. Viceversa, among the countries with large shares of senior population we find most high income countries, but also Belarus, Russia, and other middle income countries in Eastern Europe and Central Asian.

**Fig 2:**
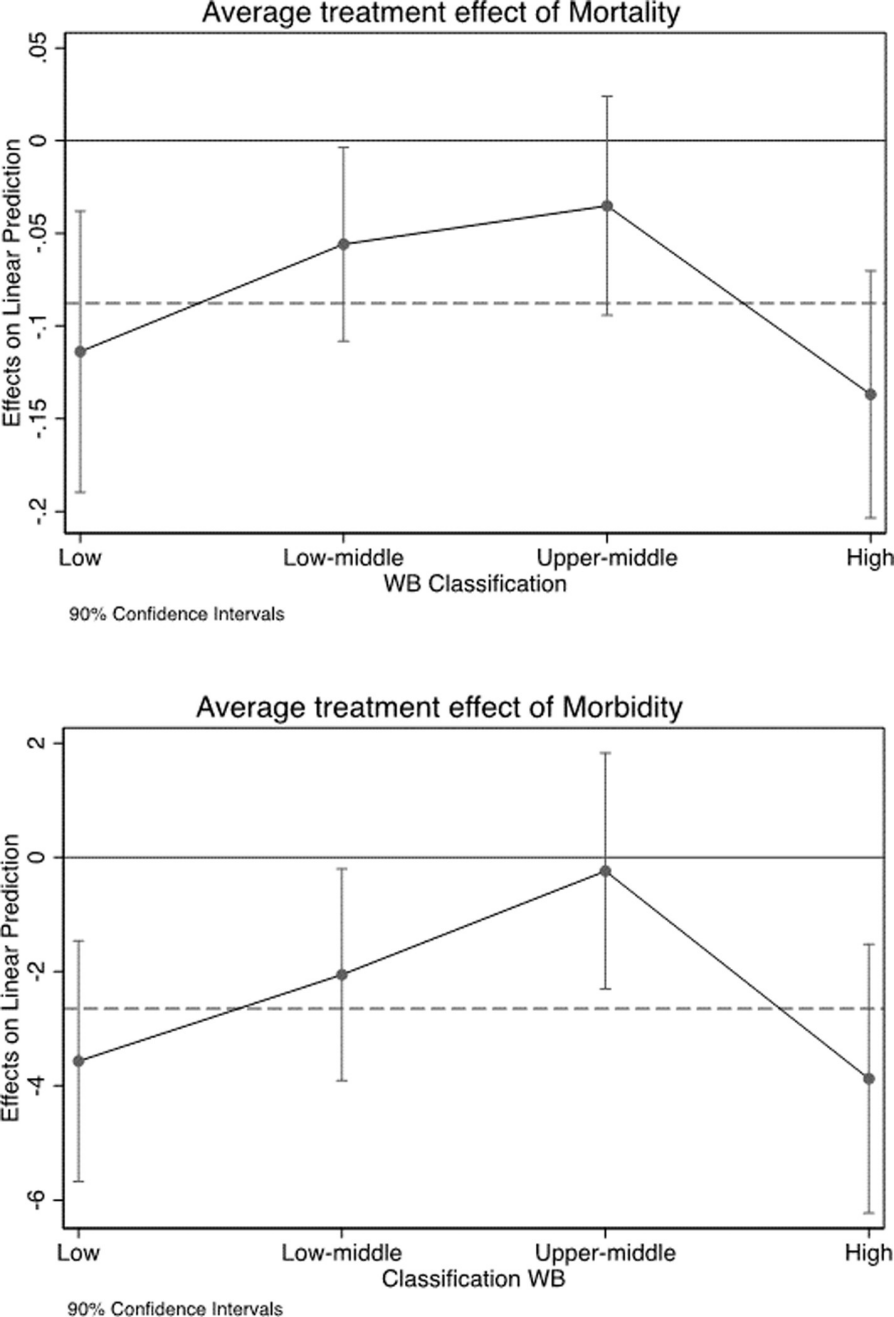
Heterogeneous effects by WB classification group. Note: Average treatment effects by WB classification group. Average treatment effects for the full sample (dotted line).

**Fig 3:**
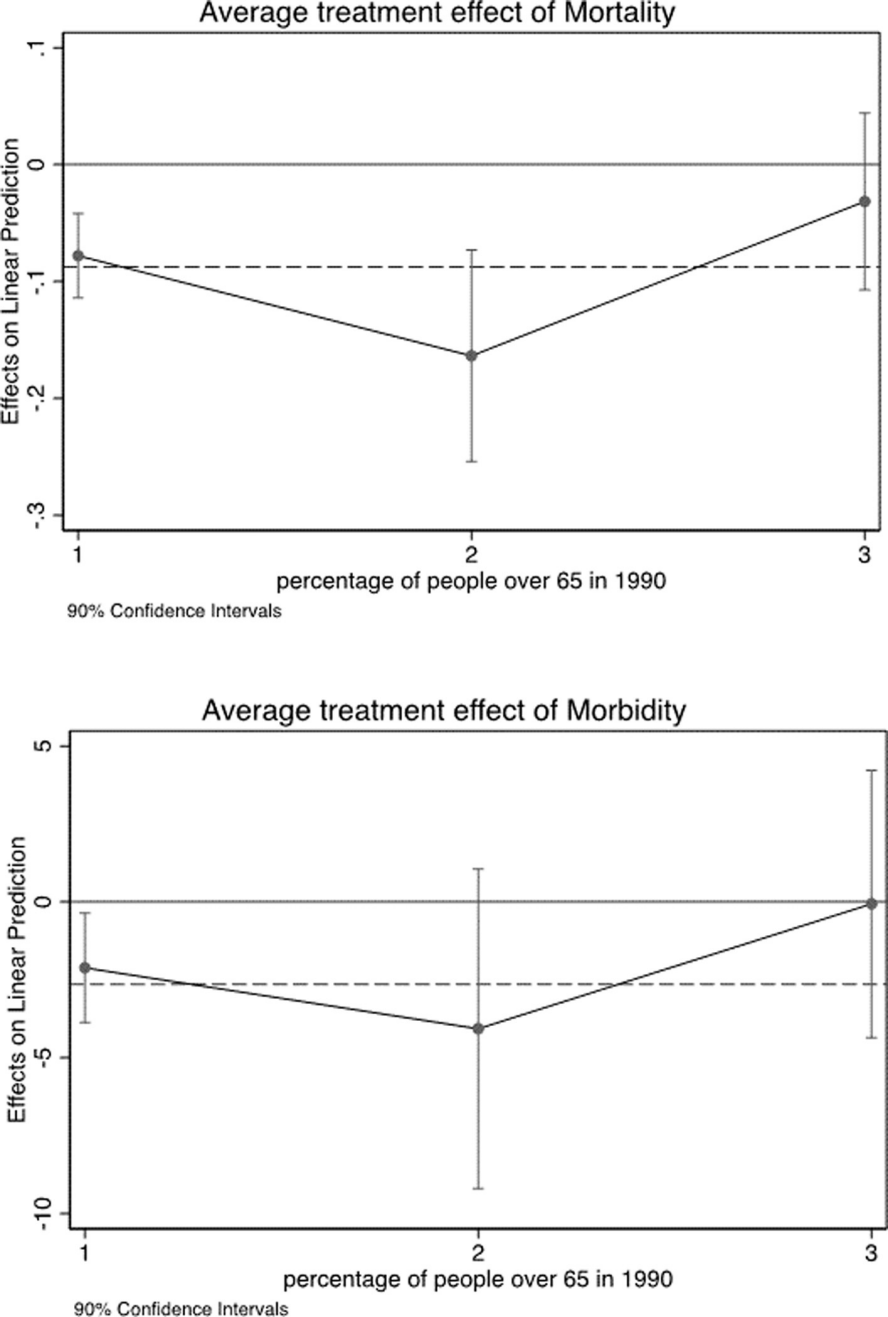
Heterogeneous effects by WB percentage of people over 65 in 1990. Note: Average treatment effects for countries whose percentage of people over 65 in 1990 is below 5% (group 1), above or equal 5% and below 10% (group 2), and 10% or more (group 3),. Average treatment effects for the full sample (dotted line).

Including interactions of ${\bar{R}}_{i}$ with indicators of country groups makes model specifications flexible and avoids assuming any functional form for the pattern of effects across countries. Leveraging on the previous analysis, which points out that OLS estimates are, if anything, conservative, and given the larger precision of OLS compared to the IV estimator, the heterogeneity analysis is conducted by OLS. Results are reported in Figs 2 and 3 and Tables A3 and A4 in appendix.

In both cases we do observe non linear patterns. The effects of mortality and morbidity tend to be stronger in the poorest and the richest countries than in the middle-income countries. Moreover, the effect vanishes in the countries with a proportion of senior population larger than 10 percent. These results imply that reducing mortality is more beneficial in countries at an earlier stage of demographic transition, supporting the mechanism proposed by [6], but also in those rich countries whose population is relatively older, but enjoys high levels of human capital, which in turn supports seniors’ labour productivity.

## 6. The macroeconomic effects of investing to reduce mortality and morbidity

To illustrate the relevance and magnitude of our findings, we compute the net present value of the flow of differential income per capita generated as a result of a marginal reduction (resp. increase) in mortality and morbidity. This back-of-the-envelope calculation might help governments evaluate the macroeconomic benefit (resp. costs) of investment decisions in health policies effective in reducing mortality and morbidity. The macroeconomic payoff of improving population health adds to the human and welfare benefits that have been discussed for instance by [28].

We consider four countries as case studies. We chose one country for each category of the 2014 World Bank income group classification: the US (high income), China (upper-middle income), India (lower-middle income) and Niger (low income). Perhaps optimistically, we assume that these four countries will be able to replicate the same reduction in mortality rates and DALYs in future periods (i.e. the period between 2015 and 2039), which they experienced in the previous quarter century (1990–2014). Between 1990 and 2014, the change in mortality (DALY) was -5.3 percent (+1.39 percent) in the US, -23.43 percent (-8.91 percent) in China, -23.74 percent (-9.88 percent) in India, and -9.67 percent (-12.1 percent) in Niger. Since we interpret the replication of these dynamics as the result of mere continuation of past health policies, we name this the *status quo scenario*.

We consider two alternative scenarios, the *positive action scenario*, and the *negative action scenario* which assume that countries step up (resp. step down) their health policy efforts and hence achieve an additional reduction (resp. increase) in mortality and DALYs by 1 percentage point, compared to the status quo. For instance, the US will experience a reduction in mortality of 6.3 percent in the positive action scenario and a reduction of 4.3 percent in the negative action scenario, compared to the 5.3 percent in the status quo scenario while, in Niger, the fall in DALYs will be 13.1 percent in the positive action scenario and 11.1 in the negative action scenario, rather than 12.1 percent in the status quo.

**Fig 4:**
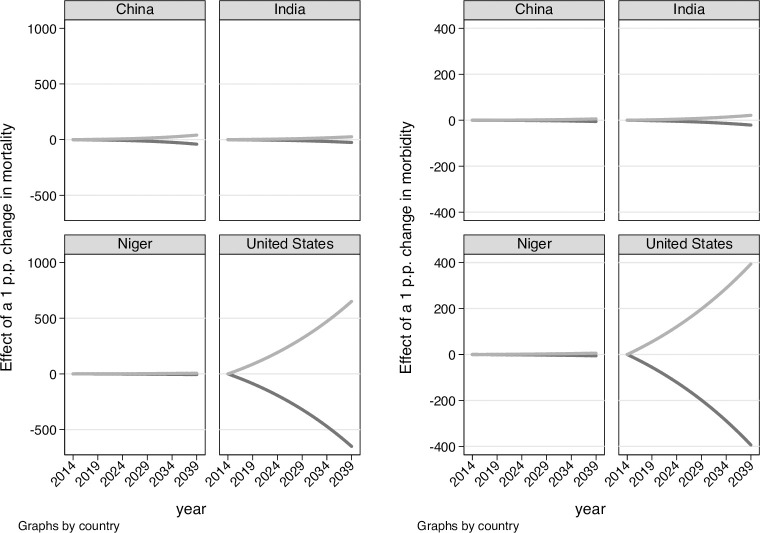
Differential GDP per capita under the action scenario compared to the status quo between 2015 and 2039. By year and country. Note: left panel: mortality; right panel: DALY. Positive action scenario in gray and negative action scenario in black.

We compute the economic benefit of such actions by comparing the dynamics of GDP per capita between 2014 and 2039 under the status quo and the two action scenarios, predicted on the basis of our (conservative) OLS estimates. We assign to each country the estimate corresponding to its income category, as reported in Table A3. Fig 4 reports the differential GDP per capita accruing under the actions scenario by year and country, while Table 5 reports the net present value (applying a 2 percent yearly discount rate) of the action scenarios, and how such figures compare with GDP per capita in 2014. The effect of the positive and negative action scenarios are practically symmetric. Larger benefits are experienced by Niger and USA, given the stronger effect of mortality and morbidity on long run growth. The differential (macro)economic value of the action scenarios compared to the status quo is 5.1 percent of 2014 GDP per capita in Niger and 10 percent in the US. Turning to DALYs, the value of the action scenarios is respectively 4.8 and 6.1 percent of 2014 GDP per capita in Niger and the US respectively. These figures would be proportionally larger if the action scenarios diverged of more than one percentage point from the status quo. Especially as regards mortality, we judge the differential economic payoff of the action scenarios as moderate but non-negligible.

**Table 5:** GDP per capita gains due to a decrease/increase in mortality (morbidity) between 2015 and 2039 1 p.p. bigger than that between 1990 and 2014.

	Mortality: Net present value of the positive action scenario	Mortality (positive action): % of the 2014 GDP	Mortality: Net present value of the negative action scenario	Mortality (negative action): % of the 2014 GDP
Niger	45.6	5.1	-45.6	-5.2
India	167.8	3.0	-167.9	-3.0
China	244.3	2.0	-244.4	-2.0
USA	5140.4	10.0	-5151.4	-10.0
	Morbidity: Net present value of the positive action scenario	Morbidity (positive action): % of the 2014 GDP	Morbidity: Net present value of the negative action scenario	Morbidity (negative action): % of the 2014 GDP
Niger	42.7	4.8	-42.7	-4.8
India	142.0	2.6	-142.0	-2.6
China	34.7	0.3	-34.7	-0.3
USA	3173.5	6.1	-3177.9	-6.2

Note: Net present value of the additional flow of income per capita accruing under the action scenario, compared to the status quo scenario, between 2014 and 2039. Projections based on the estimates reported in Table 4. Yearly discount rate: 2 percent.

## 7. Conclusion

We have thoroughly reconsidered the role of mortality and morbidity as determinants of economic growth, thereby contributing to a long-standing debate that had been fueled by influential studies reaching contrasting results. Our approach is close to LMW but it extends the analysis in three respects. First, it uses more recent and internationally comparable data on mortality and DALYs, the latter being a composite measure of mortality and morbidity that, to the best of our knowledge, has not previously been considered in the health and growth literature. Second, the sample of countries is much larger than in past studies and, third, acknowledging the difficulties of the empirical analysis, we provide bounding sets for the effects of interest rather than point estimates.

We find that reducing mortality and morbidity is beneficial to long run economic growth. Specifically, reducing mortality (resp. DALYs) by 10 percent increases the growth in GDP per capita over a quarter century by *at least* 9.6 (resp. 10.6) percentage points on average (upper bound derived according to the Nevo-Rosen procedure). Conley’s and Lewbel strategies suggest that our IV estimates are robust to violations of the exclusion restriction.

We also find that the effects are rather heterogenous and they are driven by low income and high-income countries, among which the effect is higher in absolute value than that in middle income countries, for both mortality and DALYs (See Table A3). Similarly, we find non-linear patterns across countries depending on the stage of the demographic transition they are in at the beginning of the study period. Generally, the effects are smaller in absolute terms in countries where the share of the senior population exceeds 10 percent.

Finally, we use our findings to estimate the net present value of investing more or less resources on health policies to accelerate (resp. slow down) mortality and morbidity declines in four representative countries, the US, China, India and Niger. We compute the (macro)economic benefit (resp. costs) under two hypothetical action scenarios, where the long run change in mortality and DALYs are increased (resp. reduced) by 1 percentage point during the period 2015–2039 compared to a status quo scenario. For mortality, the economic benefit of the positive action scenario and the economic cost of the negative action scenario are practically symmetric and turn out to be higher in the US and Niger (10 percent and 5.1 percent of their 2014 per-capita GDP) and lower in China and India (2 percent and 0.3 percent of the 2014 per capita GDP respectively). Figures for DALY are smaller, but they reflect the use of more conservative estimates of the effect of morbidity on economic growth. These figures suggest that the economic growth payoff from investments to improve health could be an additional factor to consider in the cost-benefit analyses of large-scale, population-level health policies.

## Appendix: Additional tables

**Table A1:** The effect of mortality and DALY on GDPpc growth. Long-Run Growth Regression (full specification reported).

	OLS	IV	OLS	IV
All-Cause mortality rate (per 100,000 inhabitants)	-0.090***	-0.096***		
	(0.019)	(0.037)		
All-Cause DALY (per 100 inhabitants)			-2.648***	-5.687**
			(0.873)	(2.488)
Log real GDPpc 1990	-43.181***	-43.717***	-34.436***	-32.783***
	(11.079)	(10.314)	(10.223)	(9.668)
OECD 1990	-46.974**	-47.833**	-40.492*	-46.509**
	(20.938)	(19.191)	(21.861)	(20.731)
Log population	19.837***	19.608***	21.676***	20.207***
	(4.379)	(5.320)	(4.556)	(5.591)
Urban	-0.569	-0.594	-0.432	-0.661
	(0.368)	(0.471)	(0.384)	(0.514)
Openness	0.517***	0.514***	0.569***	0.583***
	(0.142)	(0.120)	(0.149)	(0.135)
Government spending	-1.528***	-1.519***	-1.616***	-1.576***
	(0.410)	(0.224)	(0.429)	(0.291)
Civil liberties	-5.150	-4.998	-7.145	-7.043
	(4.209)	(4.413)	(4.388)	(4.797)
Internet	1.337*	1.275*	1.552*	0.824
	(0.784)	(0.707)	(0.826)	(0.880)
% area in polar zone	-108.569	-109.373	-105.286	-114.112
	(110.799)	(104.593)	(116.170)	(125.825)
% area in boreal zone	89.851*	91.663***	86.926*	111.945***
	(46.388)	(32.436)	(48.846)	(40.799)
% area in dry temperate zone	59.380*	61.097*	61.632*	91.109*
	(33.323)	(31.982)	(35.557)	(46.869)
% area in wet temperate zone	55.570*	57.603*	48.644	72.543**
	(30.107)	(29.485)	(31.689)	(35.738)
% area in subtropical zone	38.709*	40.223*	42.568*	70.702**
	(22.649)	(21.213)	(24.647)	(33.327)
% area in tropical zone	-10.743	-9.265	-23.852	-15.748
	(26.028)	(21.543)	(27.007)	(23.127)
Population older than 65	2.442	2.901	-3.735	-3.644
	(3.106)	(3.661)	(2.934)	(2.627)
Share of population with secondary education	0.227	0.226	0.314	0.393
	(0.325)	(0.302)	(0.342)	(0.374)
Share of population who trust other	0.634	0.658	0.579	0.890
	(0.530)	(0.657)	(0.558)	(0.722)
Missing trust	19.766	20.421	16.684	23.412
	(14.594)	(20.789)	(15.290)	(22.261)
Constant	141.961	153.517	36.864	97.217
	(109.250)	(96.037)	(110.234)	(102.057)
Semi-elasticity	-0.802	-0.861	-0.493	-1.060
R^2^	0.498		0.448	
F		58.07		24.535
Observations	135	135	135	135

Note: Robust standard errors in parentheses.

*** p<0.01,

** p<0.05,

* p<0.1. Semi-elasticity is defined as the effect on economic growth (measured as percentage points) of reducing mortality by 1 percent.

**Table A2:** The effect of mortality/DALYs on GDPpc growth. Long-Run Growth Regression (standard errors clustered at the regional level and bootstrapped using wild bootstrapping).

	(1) OLS (s.e clustered at the regional level)
All-Cause mortality rate (per 100,000 inhabitants)	-0.090*
	(0.022)
Semi-elasticity	-0.802
R^2^	0.4978
All-Cause DALYs (x 100 inhab.)	-2.648**
	(1.017)
Semi-elasticity	-0.493
R^2^	0.4479
Observations	135

Note: Wild bootstrapped cluster-robust standard errors in parentheses.

*** p<0.01,

** p<0.05,

* p<0.1. Controls as in Table A1. Semi-elasticity is defined as the effect on economic growth (measured as percentage points) of reducing mortality by 1 percent.

**Table A3:** The effect of mortality/DALYs on GDPpc growth. Long-Run Growth Regression. Effects by World Bank Country Income Group. OLS.

	Low GDP countries	Middle Low GDP countries	Middle High GDP countries	High GDP countries
All-Cause mortality rate (per 100,000 inhabitants)	-0.114***	-0.056**	-0.035	-0.137 ***
	(0.038)	(0.026)	(0.030)	(0.034)
Semi-elasticity	-1.020	-0.501	-0.316	-1.227
R^2^	0.6671	0.6671	0.6671	0.6671
All-Cause DALYs (x 100 inhab.)	-3.567***	-2.054*	-0.241	-3.874***
	(1.268)	(1.119)	(1.245)	(1.418)
Semi-elasticity	-0.665	-0.382	-0.045	-0.722
R^2^	0.6248	0.6248	0.6248	0.6248
Observations	135	135	135	135

Note: Robust standard errors in parentheses.

*** p<0.01,

** p<0.05,

* p<0.1. Controls are: Log real GDP per capita 1990, share of the population aged 65 and over in 1990, OECD 1990, log population, dummy indicator of rural areas, government spending, civil liberty index, percentage of household with internet access, share of population with at least secondary education, share of population reporting their trust the others in the WVS, dummy indicating whether the trust variable has been imputed, % of country area in polar zone, % of country area in boreal zone, % of country area in temperate zone, % of country area in subtropical zone, % of country area in tropical zone. Semi-elasticity is defined as the effect on economic growth (measured as percentage points) of reducing mortality by 1 percent at the sample mean.

**Table A4:** The effect of mortality/DALYs on GDPpc growth. Long-Run Growth Regression. Effects by Percentage of people over 65. OLS.

	Percentage of people above 65 smaller than 5%	Percentage of people above 65 between 5% and 10%	Percentage of people above 65 bigger than 10%
All-Cause mortality rate (per 100,000 inhabitants)	-0.078***	-0.164***	-0.032
	(0.022)	(0.055)	(0.046)
Semi-elasticity	-0.699	-1.466	-0.283
R^2^	0.5417	0.5417	0.5417
All-Cause DALYs (x 100 inhab.)	-2.112**	-4.067	-0.064
	(1.055)	(3.089)	(2.584)
Semi-elasticiy	-0.394	-0.758	-0.012
R^2^	0.4906	0.4906	0.4906
Observations	135	135	135

Note: Robust standard errors in parentheses.

*** p<0.01,

** p<0.05,

* p<0.1. Controls are: Log real GDP per capita 1990, share of the population aged 65 and over in 1990 (3 dummies), OECD 1990, log population, dummy indicator of rural areas, government spending, civil liberty index, percentage of household with internet access, share of population with at least secondary education, share of population reporting their trust the others in the WVS, dummy indicating whether the trust variable has been imputed, % of country area in polar zone, % of country area in boreal zone, % of country area in temperate zone, % of country area in subtropical zone, % of country area in tropical zone. Semi-elasticity is defined as the effect on economic growth (measured as percentage points) of reducing mortality by 1 percent at the sample mean.

**Table A5:** The effect of mortality/DALYs on GDPpc growth. Long-Run Growth Regression. Effects by alternative measures of mortality and morbidity.

	OLS	IV	OLS	IV
All-Cause mortality rate (per 100,000 inhabitants), age 15–64	-0.078***	-0.132**		
	(0.027)	(0.055)		
All-Cause DALY (per 100 inhabitants), all ages			-1.579***	-1.501***
			(0.342)	(0.551)
Observations	135	135	135	135
Semi-elasticity	-0.377	-0.636	-0.708	-0.673
F		33.201		50.278

Note: Robust standard errors in parentheses.

*** p<0.01,

** p<0.05,

* p<0.1. Controls are: Log real GDP per capita 1990, share of the population aged 65 and over in 1990, OECD 1990, log population, dummy indicator of rural areas, government spending, civil liberty index, percentage of household with internet access, share of population with at least secondary education, share of population reporting their trust the others in the WVS, dummy indicating whether the trust variable has been imputed, % of country area in polar zone, % of country area in boreal zone, % of country area in temperate zone, % of country area in subtropical zone, % of country area in tropical zone. Semi-elasticity is defined as the effect on economic growth (measured as percentage points) of reducing mortality by 1 percent at the sample mean.
